# Application of tea polyphenols in combination with 6-gingerol on shrimp paste of during storage: biogenic amines formation and quality determination

**DOI:** 10.3389/fmicb.2015.00981

**Published:** 2015-09-16

**Authors:** Luyun Cai, Shucheng Liu, Lijun Sun, Yaling Wang, Hongwu Ji, Jianrong Li

**Affiliations:** ^1^Key Laboratory of Aquatic Product Processing and Safety of Guangdong Province, College of Food Science and Technology, Guangdong Ocean UniversityZhanjiang, China; ^2^Food Safety Key Lab of Liaoning Province, National and Local Joint Engineering Research Center of Storage, Processing and Safety Control Technology for Fresh Agricultural and Aquatic Products, College of Food Science and Engineering, Bohai UniversityJinzhou, China

**Keywords:** tea polyphenols, 6-gingerol, shrimp paste, biogenic amine, quality

## Abstract

Tea polyphenols (TP) have shown antioxidant activity and antimicrobial properties in the food industry. Assessment of anti-oxidation potential of 6-gingerol (GR) has also been verified. As little is known about the use of tea polyphenols either individually or in combination with 6-gingerol in shrimp paste, we aimed to investigate the effect of tea polyphenols combined with 6-gingerol on the biogenic amines inhibition and quality of shrimp paste stored at 25°C for 160 days. The shrimp paste samples were assigned into four groups: (1) control; (2) tea polyphenols treatment (0.3%); (3) 6-gingerol treatment (0.3%); (4) tea polyphenols (0.15%) + 6-gingerol (0.15%). Samples with no addition were used as control. The results indicate that treatment with tea polyphenols + 6-gingerol (TPGR) maintained paste appearance, inhibited oxidation of protein and lipids, and reduced microorganism counts compared to control treatment. The efficiency was superior to that of tea polyphenols or 6-gingerol treatment. Furthermore, shrimp paste treated with TPGR also exhibited significantly higher inhibition of biogenic amines. Total amino acids determination proved the efficacy of TPGR by maintaining the more amino acids of shrimp paste during ambient temperature storage. Our study suggests that TPGR might be a promising candidate for fermented foods due to its synergistic effect to maintain products quality and extending their shelf-life.

## Introduction

Shrimp fermentation is one of the most common methods of shrimp preservation in Asia countries due to the simplicity of technology and low cost of equipment. Shrimp paste is one of the most popular products in the eastern coastal areas of China due to its salty, rich seafood flavor and characteristic appetite-stimulating aroma. Shrimp paste is traditionally obtained through the natural fermentation process of whole shrimp in the presence of salt (25–30% on the weight basis) under ambient conditions. Although the traditional shrimp paste is insusceptible to contamination by microorganisms and has long shelf life of 6 months under room temperature conditions, it is usually used as condiments due to its high concentration of salt. Low-salt shrimp paste is also obtained by the fermentation with small shrimp used as raw materials. It satisfies the public demands for healthy food owing to its high moisture and low salinity (≤ 18%), while lower salinity may decrease the antimicrobial capacity and thus affecting the quality of products stored at room temperature, resulting in short shelf life of about 100 days. The short shelf life of low-salt shrimp paste is an impediment to the distribution and marketing of the room temperature products. Thus, prolonging the storage period, while preserving their quality, would benefit the shrimp paste industry as well as consumers.

Several recent studies have focused on using natural ingredients to enhance products quality during storage (Feng et al., 2012; Li et al., 2013; Cai et al., 2014, 2015a). Tea polyphenols (TP) have shown antioxidant activity and non-toxicity in the food industry. TP has also been proved to be effective against cancer and cardiovascular disease. Thus, TP has beneficial prospects for its use as antioxidants (Kuriyama et al., 2006; Pasrija et al., 2015). The antioxidant mechanism of polyphenols is mainly due to their capacity in scavenging reactive oxygen radicals and chelating metal ions (Dangles, 2012; Afzal et al., 2015). Additionally, tea polyphenols also possess antimicrobial properties (Chinnathambi and Alharbi, 2013). Ginger (*Zingiber officinale* Roscoe) is one of the commonly used spices belonging to the Zingiberaceae family and widely used in processed food, such as chutneys, jams, pickles, beverages and bakery products, as well as in other industrial sectors. It is regularly used as seasonings to enhance the sensory quality of food. Besides researching its health benefits (Shariatpanahi et al., 2010), phytochemicals obtained in ginger and their antimicrobial activities against some microorganisms were also investigated. 6-Gingerol (GR) extracted from rhizome of the ginger is reported to possess various bioactive properties such as anticancer, anti-inflammation, antimicrobial, and anti-oxidation (Singh et al., 2008; Baliga et al., 2012; Yusof et al., 2015). In particular, 6-gingerol could reduce bacterial biofilm formation and virulence via quorum sensing inhibition (Kim et al., 2015). Assessment of anti-oxidation potential of 6-gingerol has also been verified, which makes it important to apply it in pharmaceutical, agronomic, and food industries, as food preservers and additives and as natural remedies (Jeena et al., 2014).

However, to our knowledge, the use of tea polyphenols either individually or in combination with 6-gingerol, has not been studied to date, in shrimp paste. Thus, the objective of the present study was to determine the effect of tea polyphenols and 6-gingerol, applied individually and/or in combination, on quality of shrimp paste during room temperature storage, to further understand the roles of tea polyphenols and 6-gingerol as antioxidants and antimicrobials during the storage of shrimp paste.

## Materials and methods

### Samples treatment and chemicals

The shrimp (Acetes spp.) was cleaned and filtered with a layer of nylon screen. The washed shrimp was filtered, followed by coarse grinding using basket centrifuge and autoclave at 115°C for 10 min. The starter was prepared by fermenting the mixture of flour (10%, w/w) and starter culture (0.1%, w/w) and distilled water (10%, w/w) at 32°C for 20 h. To exhibit the characteristic and amazing taste of shrimp paste, the mixture was fermented by adding salt (18%, w/w) at 42°C for 30 d. The paste was grinded by colloid mill and sterilized at 121°C for 15 min. In a preliminary experiment, we measured a series of concentrations of both additions, including tea polyphenols and 6-gingerol, that is, 0.2, 0.3, 0.4, and 0.5%. All natural ingredients at the concentration of 0.2 or 0.3% significantly inhibited paste spoilage, and 0.3% had the preferable effect. However, 0.4 or 0.5% of treatment caused some sensory damages, including off-flavor or discoloration in the shrimp paste. Therefore, a concentration of 0.3% was chosen to use in this experiment. The Shrimp paste samples were randomly assigned into four groups: (1) control; (2) tea polyphenols treatment (0.3%); (3) 6-gingerol treatment (0.3%); (4) tea polyphenols (0.15%) + 6-gingerol (0.15%). Samples with no addition were used as control.

After that, they were agitated by a magnetic stirrer for 10 min. For each group, 30 pieces of shrimp paste were used. Then, the shrimp paste samples were stored at 25 ± 1°C for subsequent quality assessment. Physicochemical, biogenic amines, microbiological and sensory analyses were performed at 40-day intervals to determine the quality of shrimp paste. Tea polyphenols were purchased from Zhejiang University Tea Scientific., Ltd (purity ≥98%, Hangzhou, China). 6-gingerol was obtained from Xian Changyue Phytochemistry Co., Ltd. (Purity ≥95%, Xian, China). All chemicals were of analytical grade.

### Color measurement

The color of shrimp paste samples (10 g) were measured with a WSC-S colorimeter (Shanghai Precision Instrument Co. Ltd., Shanghai, China). Data collected included color coordinates lightness values (L^*^), a^*^ values (red-green scale) and b^*^ values (yellow-blue scale). The color intensity is expressed by the chroma value (C^*^ab), while hue (H^0^ab) represents the purity of color, were respectively calculated according to the formula: C^*^ab = (a^*2^+b^*2^)1/2 and H^0^ab = arctan (a^*^/b^*^).

### Total volatile basic nitrogen (TVB-N)

The TVB-N values were determined as described by Ozogul and Balikci (2013) with a Kjeltec 8400 (Foss, Sweden) using steam distillation for extraction volatile bases from shrimp paste samples. Briefly, 10 g of shrimp paste was homogenized with 50 mL of distilled water on a Kjeldahl distillation tube. After homogenization, 3 mL of silicone anti-foaming agent and 1 g of MgO were added. The distillate was collected into 10 mL of 0.1 M hydrochloric acid solution with an indicator solution (methyl red). Steam distillation process was ended after check with a pH strip for the complete absence of alkalinity on the distillate. The distillate was titrated with 0.0167 M sodium hydroxide solution, and the results were expressed in mg nitrogen per 100 g sample.

### Thiobarbituric acid reactive substances (TBARS)

In this study, the TBA value of shrimp paste samples was evaluated by measuring the concentration of malonaldehyde (Botsoglou et al., 1994) with some modification. Samples (200 mg) were homogenized with 4.8 mL of a 5% solution of potassium chloride. To 0.5 mL of homogenate, 3 mL of 1% phosphoric acid and 1 mL of 0.6% TBA aqueous solution were added. The mixture was incubated in boiling water for 90 min followed by an ice bath for 10 min. Then 4 mL of 1-butanol was added. The tubes were shaken and the supernatant was removed after centrifugation. The absorbance (As) of the resulting pigment was recorded at 532 nm using a UV-Vis spectrophotometer (UV-2550, Shimadzu). A reagent blank was run and the absorbance (Ab) recorded. The absorbance values were converted to the TBA value (mg of malonaldehyde equivalents/kg of tissue) using Equation (1):

(1) TBA=50×(As−Ab)/200

### Total amino acids composition

The total amino acids contents were determined using a full-automatic amino acid analyzer (L-8900A, Hitachi, Tokyo, Japan). An appropriate pretreatment for the samples was necessary before amino acids analysis, according to the method proposed by Kim et al. (2003) with some modifications. Samples (10 mg) were hydrolyzed in 6 M HCl in evacuated sealed tubes at 110°C for 24 h. A calibration curve was obtained with standard amino acid mixture (Sigma, St. Louis, MO, USA) and qualitative analysis was made on the basis of retention time and peak area of standard compounds.

### Microbiological analyses

Shrimp paste samples (10 g) were diluted with phosphate buffer (3.4% w/v, pH 7.2) in sterile containers to an initial dilution of 1:10. Additional serial dilutions were performed when needed. Total viable counts (TVC) was determined on plate count agar (PCA, Aoboxing Bio-Tech, Beijing, China) by counting the number of colony-forming units after incubation at 35°C for 48 h. Three replicates were made for each sample and four appropriate dilutions were used for each replicate. Microbiological data were transformed into logarithms of the number of colony forming units (CFU/g).

### Biogenic amines

BAs of all samples were determined according to the methods described by Park et al. (2010). Briefly, 5 g of each sample were homogenized with 20 ml 0.1 M hydrochloric acid using a homogenizer for 1 min. The homogenate was centrifuged at 11,190 g for 15 min, and the supernatant was collected. The residue was extracted again with 20 ml 0.1 M hydrochloric acid. The supernatants were then combined and adjusted to 50 ml with 0.1 M hydrochloric acid. A stock of standard solution was prepared by adding an accurately weighed amount of each amine (100 mg) to a 100 ml volumetric flask and brought to the mark with 0.1 M HCl. The standard solutions were stored at 4°C until use. Each extracted sample or standard solution (0.3 ml) was mixed with 0.05 ml of saturated NaHCO_3_ and 0.05 ml of 2 M NaOH. 0.3 ml of 10 mg/ml DNS-Cl solution prepared in acetone was added and the reaction mixture was incubated at 45°C for 1 h in darkness. Residual DNS-Cl was removed by adding 0.02 ml 25% ammonia. After 30 min the mixture was adjusted to 1.0 mL with acetonitrile and centrifuged at 2417 g for 10 min. The supernatant was filtered through 0.22-μm filters prior to HPLC analysis.

The quantification of BAs was carried out using an optimized reverse-phase HPLC (Agilent, 1260 LC) equipped with a Agilent C18 (5 μm, 4.6 × 250 mm) column and a UV detector (Agilent, 1260 LC). Distilled water (solvent A) and acetonitrile (solvent B) were used as mobile phases. Elution was carried out using the following gradient: 0 min, 55% B; 15 min, 65% B; 20 min, 80% B; 30 min, 90% B; 35 min, 55% B. The sample (10 μl) was injected at a flow rate of 1 ml/min and the peaks were detected at 254 nm.

### Sensory evaluation

Sensory evaluation of shrimp paste (10 g) was performed by an 8 trained panel using the structured scaling test. Panel development followed the prescreening, screening, training, and performance evaluation phases as described previously (Cross et al., 1978). Panelists scored for color, aroma, flavor and juiciness of shrimp paste according to a 9-point hedonic scale (9—like extremely to 1—dislike extremely). A sensory score of 4 was taken as the borderline of acceptability.

### Statistical analysis

The experiment followed a completely randomized design (*n* = 3). Data were subjected to One-Way analysis of variance (ANOVA). Mean separations were assessed by Duncan's multiple range test (SAS Version 8.1). Differences at *p* < 0.05 were considered significant.

## Results and discussion

### Effect of tea polyphenols in combination with 6-gingerol treatment on color

The appearance of food products is of major importance to consumers, both from the point of view of acceptability and of preference. So, color plays a crucial role when evaluating the quality of the shrimp paste at the point of sale. Different values obtained after application of TPGR, compared to the control treatment, are shown in Table 1. From this table, the L^*^ values of control samples significantly (*p* < 0.05) decreased after 40 days, and it was 41.77 at day 80 and 38.64 at day 120; higher L^*^ values were observed in TPGR samples compared to control after 40 days; the TPGR samples were higher than the TP or GR samples in L^*^ values, mainly attributed to the inhibition of 6-gingerol on melanosis formation in shrimp paste and also the synergistic effect of TP combined with GR treatment (Stoilova et al., 2007; Nile and Park, 2015). The values of b^*^ decreased from day 0 to day 160, indicating an evolution toward gray tones as the storage time, and the similar decreasing trend was found in the values of a^*^. Regarding parameters C^*^ab and *H*^0^*ab*, no significant differences were observed in TPGR samples compared to TP or GR samples in the present study.

**Table 1:** **Changes in color of shrimp paste treated with tea polyphenols + 6-gingerol stored at 25°C for 160 days**.

**Treatments**	L^*^	a^*^	b^*^	C^*^ab	**H^0^ab**
**0 DAYS**					
Control	46.50±0.16aA	2.74±0.10aA	17.51±0.85aA	17.72±0.85aA	0.16±0.01aA
TP	46.37±0.68aA	2.61±0.12aA	17.77±0.54aA	17.96±0.55aA	0.15±0.01aA
GR	47.25±0.47aA	2.64±0.19aA	17.67±0.61aA	17.87±0.64aA	0.15±0.01aA
TPGR	47.10±1.20aA	2.78±0.06aA	17.72±0.55aA	17.94±0.56aA	0.16±0.01aA
**40 DAYS**					
Control	44.48±0.84bA	2.29±0.16bB	16.31±0.91aAB	16.47±0.92aA	0.14±0.01aA
TP	44.97±0.68abB	2.36±0.10abB	17.10±0.46aAB	17.26±0.47aAB	0.14±0.01aA
GR	44.94±0.80abB	2.42±0.10abA	17.34±1.31aA	17.51±1.32aAB	0.14±0.01aA
TPGR	46.57±1.10aAB	2.57±0.14aA	17.47±0.15aA	17.66±0.20aAB	0.15±0.01aA
**80 DAYS**					
Control	41.77±1.04bB	1.62±0.13bC	14.85±0.49aB	14.94±0.50aB	0.11±0.01bB
TP	43.20±0.89bC	1.94±0.10aC	16.08±1.35aAB	16.20±1.35aAB	0.12±0.01abAB
GR	42.67±0.53bC	2.16±0.11aB	15.97±0.53aA	16.12±0.54aAB	0.13±0.01aA
TPGR	45.64±1.23aABC	1.92±0.16aB	16.17±0.71aAB	16.28±0.73aBC	0.12±0.01abB
**120 DAYS**					
Control	38.64±0.83cC	1.40±0.10bC	12.75±0.86bC	12.82±0.86bC	0.11±0.01aB
TP	41.95±0.81bC	1.69±0.14abD	15.53±1.23aB	15.62±1.24aB	0.11±0.01aB
GR	41.24±0.67bD	1.63±0.11abC	15.78±0.82aA	15.86±0.83aB	0.10±0.01aB
TPGR	44.28±1.77aBC	1.61±0.19aC	16.30±0.55aAB	16.38±0.58aABC	0.10±0.01aC
**160 DAYS**					
Control	34.97±1.70cD	0.55±0.18bD	9.47±0.84cD	9.48±0.86cD	0.06±0.02bC
TP	40.06±0.51bD	1.31±0.18aE	11.30±1.36bcC	11.38±1.37bcC	0.12±0.03aAB
GR	40.43±1.05bD	1.27±0.13aD	13.61±1.31abB	13.67±1.31abC	0.09±0.02abB
TPGR	43.93±0.60aC	1.52±0.10aC	14.82±1.55aB	14.89±1.56aC	0.10±0.01aBC

TP, tea polyphenols; GR, 6-gingerol; TPGR, tea polyphenols + 6-gingerol; C

* *ab, chroma value; H^0^ab, hue value. Values are the mean of three replications ± standard deviation. Means between the treatments with different small letters are significantly different (p < 0.05). Means as storage time with different capital letters are significantly different (p < 0.05)*.

### Effect of tea polyphenols in combination with 6-gingerol treatment on TVB-N

**Figure 1:**
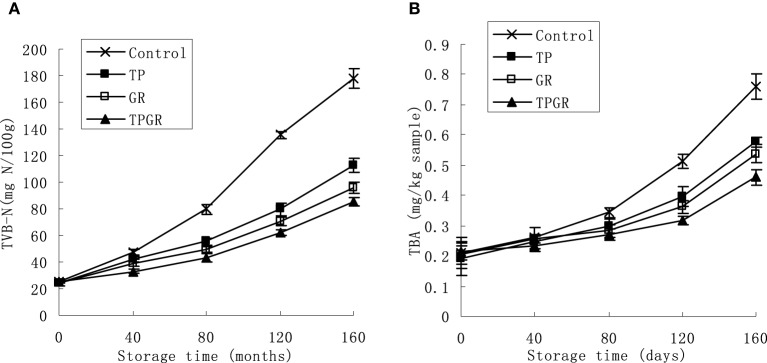
**Changes in TVB-N (A) and TBA (B) of shrimp paste treated with control (×), TP (■), GR (□), and TPGR (▲) stored at 25°C for 160 days**. Each data point is the mean of three replicate samples. Vertical bars represent standard deviation of means.

**Figure 2:**
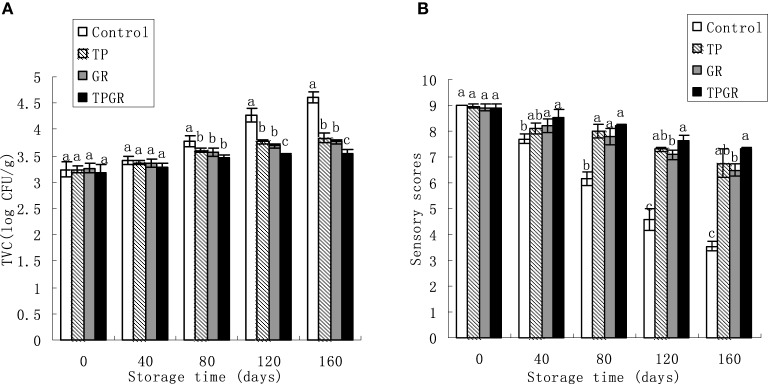
**Changes in total viable count (A) and sensory quality (B) of shrimp paste stored at 25°C for 160 days**. Each data point is the mean of three replicate samples. Vertical bars represent standard deviation of means. Different small letters indicate significant differences (*p* < 0.05) between treatments.

The TVB-N, which is mainly composed of ammonia and primary, secondary and tertiary amines, is widely used as an indicator of aquatic products spoilage. TVB-N values of shrimp paste during 25°C storage were gradually increased (Figure 1A). The increasing order of TVB-N values with different treatments at day 160 were: TPGR (85.13 mg N/100 g) < GR (95.75 mg N/100 g) < TP (113.02 mg N/100 g) < Control (178.05 mg N/100 g). Values of control samples were significantly (*p* < 0.05) higher than TP and GR treated samples. The shrimp paste samples contained TPGR had the higher effect of TVB-N inhibition (*p* < 0.05) than the TP or GR samples from day 40 to the end. The increase of TVB-N is related to the activity of spoilage bacteria (Kim et al., 2003; Cai et al., 2014). The associated addition of anti-bacterial tea polyphenols and 6-gingerol may have the intensified action on inhibiting the microbial decomposition of shrimp paste protein. Total visible counts (TVC) increased mentioned subsequently (Figure 2) during storage could explain the rise of TVB-N.

### Effect of tea polyphenols in combination with 6-gingerol treatment on TBA value

TBA values represent the content of secondary lipid oxidation products, mainly malonaldehyde (MDA), which contribute to off-flavor in oxidized foods. Figure 1B shows changes in TBA values of treated and control shrimp paste during 160 days' storage. The lipid oxidation was accelerated as storage progressed due to the enzyme released by microorganisms and aerobic storage. The initial TBA values of the control samples were 0.21 mg/kg sample and increased to 0.76 mg/kg sample after 160 days storage. TP and GR reduced the final TBA values by 23.7 and 28.9% than the control samples, respectively, and GR had the higher inhibitory effect, showing the stronger antioxidation than TP. TBA is an appropriate indicator to assess lipid oxidation due to its relatively simple measurement and correlation with the sensory quality of food. Shrimp paste treated with TPGR had the lowest TBA value (0.46 mg/kg sample) at the end of storage. This may due to strong antioxidant activity of spice extracts and synergistic effect with tea polyphenols. These results were consistent with studies done by Zhao et al. (2013) who reported that large yellow croaker immersed with tea polyphenols enhanced fish muscle quality and Cai et al. (2015b) who reported that red sea bream filets treated with 6-gingerol inhibited the increase of TBA. Additionally, Bandyopadhyay et al. (2008) indicated the synergistic effectiveness of natural antioxidants in controlling lipid oxidation in dairy dessert.

### Effect of tea polyphenols in combination with 6-gingerol treatment on total amino acids

Table 2 presents the total amino acids content of shrimp paste after 160 days' storage. Total amino acids content of 4819.60 mg/100 g of control samples decreased to 4478.24 mg at day 80 (data were not shown), but it decreased sharply from days 80 to 160. The major total amino acids present in the samples were aspartic acid, glutamic acid, glycine, valine, leucine and lysine, and most of them significantly decreased up to day 160 (*p* < 0.05). Lysine, as the highest content, which is a limited amino acid in grains such as rice, in the shrimp paste may act as a nutritional supplement. The TPGR samples had a significantly (*p* < 0.05) higher content of lysine than the TP or GR samples at the end of storage. The decline in the total amino acids content could be attributed to its degradation to amines, volatile acids, and other nitrogenous substances as by-products of bacterial metabolism or enzymatic decomposition (Izquierdo Canas et al., 2008). Additionally, the observed decline in amino acids would be also related with the formation of maillard reaction products (Faithong and Benjakul, 2014), as manifested by the deepening in color.

**Table 2:** **Changes in total amino acids of shrimp paste treated with tea polyphenols + 6-gingerol stored at 25°C for 160 days**.

**Amino acids**	**Control**		**TP**		**GR**		**TPGR**	
	**0 day**	**160 days**	**0 day**	**160 days**	**0 day**	**160 days**	**0 day**	**160 days**
Asp	477.72±13.39a	379.56±4.59c	483.14±6.76a	447.65±5.07b	482.91±10.47a	468.34±5.73a	479.30±4.72a	466.31±4.76a
Thr	214.94±2.00a	174.96±4.10c	213.94±3.11a	191.89±2.23b	213.74±5.18a	193.84±1.45b	213.99±4.40a	204.00±4.63a
Ser	159.95±5.36a	135.59±2.60b	160.38±3.58a	146.92±3.30a	161.67±4.97a	147.64±1.96a	163.49±4.52a	151.05±3.19a
Glu	381.98±7.70a	300.59±11.88b	380.85±4.51a	354.68±12.15a	384.29±7.51a	359.62±3.91a	384.31±2.57a	365.95±5.59a
Gly	427.45±8.47a	319.84±7.05c	421.83±4.03a	389.25±7.39b	420.55±7.39a	389.92±4.06b	422.48±5.80a	408.72±5.85a
Ala	249.49±7.90a	166.67±6.05c	244.11±5.50a	209.81±6.78b	248.16±5.26a	219.70±4.12b	246.18±4.33a	234.60±6.29a
Cys	35.51±2.87a	22.29±1.39c	37.72±5.78a	29.87±1.67b	35.83±1.64a	31.69±0.81ab	36.54±3.01a	33.40±1.42a
Val	421.81±3.05a	295.26±9.39c	418.80±7.30a	383.07±6.96b	419.80±4.74a	385.32±9.27b	416.80±1.75a	401.95±5.03a
Met	94.68±3.49a	61.92±3.06b	93.80±2.19a	82.53±4.10a	93.02±4.89a	78.98±4.12a	94.09±1.49a	85.34±2.51a
Ile	236.44±5.71a	178.67±4.71c	237.58±2.64a	209.28±7.46b	237.95±5.54a	208.84±4.58b	237.21±2.21a	223.58±4.71a
Leu	375.52±4.80a	308.59±3.66c	376.72±5.33a	339.62±5.35b	375.54±0.82a	347.09±4.34b	379.17±4.84a	365.33±8.40a
Tyr	113.89±8.21a	74.90±2.27b	110.53±4.56a	97.31±3.93a	111.82±7.62a	96.43±4.68a	113.74±7.80a	101.62±3.80a
Phe	338.42±5.04a	253.95±3.86d	334.46±3.26a	286.57±2.56c	337.98±9.06a	295.39±3.40b	337.51±2.84a	304.87±3.44a
Lys	610.46±5.35a	514.01±5.32c	613.77±5.49a	574.66±0.80b	612.25±6.42a	575.77±6.06b	615.54±3.54a	586.91±5.54a
His	80.11±4.79a	58.59±5.31b	79.73±4.12a	77.15±1.62a	86.26±3.55a	74.47±5.00a	81.79±3.45a	76.87±4.23a
Arg	312.37±3.50a	236.58±5.80d	312.20±6.63a	277.89±4.26c	312.51±6.15a	290.40±4.15b	314.52±2.55a	302.24±6.59a
Pro	288.86±4.41a	208.77±6.57b	286.74±6.27a	256.03±2.65a	283.34±1.65a	259.74±5.23a	286.57±2.50a	264.64±3.44a
Total	4819.60	3690.74	4806.31	4354.17	4817.62	4423.19	4823.22	4577.39

TP, tea polyphenols; GR, 6-gingerol; TPGR, tea polyphenols + 6-gingerol; C^*^ab, chroma value; H^0^ab, hue value. Values are the mean of three replications ± standard deviation. Means between the treatments with different small letters are significantly different (p < 0.05).

### Effect of tea polyphenols in combination with 6-gingerol treatment on microbiological analyses

The TVC values for the three different treatments as a function of storage time are shown in Figure 2A. The initial TVC values are in the range of 3.19–3.26 log CFU/g and gradually increased during room temperature storage. Control samples exceeded the value of 4 log CFU/g for TVC after the 120 days of storage, which was regarded as the upper microbiological limit for shrimp paste (Bueno-Solano et al., 2009), while GR and TP samples did not reach this value until 160 days of storage. At the end of the storage, the TVC of the TPGR samples was 3.55 log CFU/g, which was lower than the TP or GR samples (*p* < 0.05). It is evident from this study that tea polyphenols combined with 6-gingerol treatment was more effective in reducing microbial counts than other treatments, which was similar to the result studied by Cai et al. (2015b) who reported that alginate coating combined with 6-gingerol treatment inhibited microbial growth during the storage of red sea bream. This result could be due to the synergistic effect of tea polyphenols and 6-gingerol, which had the strong reducibility and could efficiently scavenge the free radicals (Wagner, 2011), thus, by combined application in shrimp paste to form a synergistic effect.

### Effect of tea polyphenols in combination with 6-gingerol treatment on biogenic amines

Biogenic amines are primarily produced via decarboxylation of amino acids by substrate-specific enzymes generated by microorganisms found in fermented foods. The type and amount of biogenic amines formed during storage depends on many factors, such as shrimp species, microbial flora, packaging, temperature and use of antimicrobial agents (Moon et al., 2010). Table 3 shows the effects of different treatments on biogenic amines in the samples throughout the storage time. The initial shrimp paste had low biogenic amines level (< 50 mg/kg), which was within the safe level for human health. Histamine is the causative agent for shrimp products poisoning, the toxic effects of which are intensified by the presence of other amines, such as putrescine and cadaverine. The highest histamine concentration was observed in the control samples (113.37 mg/kg), and lower level of histamine in TP or GR samples suggested that tea polyphenols inhibited the growth of bacteria with histidine decarboxylase activity. Kumudavally et al. (2008) reported the effects of tea polyphenols on biogenic amines formation in mutton stored at ambient temperature. They found that tea polyphenols had potentially inhibition effects on cadaverine and other BAs accumulation in mutton muscle. Cai et al. (2015a) studied that red drum filets treated with spice essential oils inhibited the formation of histamine, putrescine and cadaverine. Similarly, the contents of putrescine, histamine and tyramine were reduced compared to control in salted and fermented anchovy treated with garlic and other spices extracts, respectively, showing the higher inhibitory capacity (Mah et al., 2009). In brief, tea polyphenols combined with 6-gingerol did significantly (*p* < 0.05) inhibit the formation of histamine in shrimp paste during storage. These could be attributed that both tea polyphenols and 6-gingerol have antimicrobial and antioxidant properties. Tyramine was not detected at the beginning of storage period, and TPGR had the greatest inhibitory effects on tyramine, tryptamine and phenlethylamine in shrimp paste. Spermidine and spermine were not detected in samples throughout the storage period.

**Table 3:** **Changes in biogenic amines of shrimp paste treated with tea polyphenols + 6-gingerol stored at 25°C for 160 days**.

**BAs**	**Treatments**	**Days**				
		**0**	**40**	**80**	**120**	**160**
PUT	Control	5.13±0.38aE	15.39±2.81aD	31.07±2.51aC	55.14±2.42aB	78.20±2.88aA
	TP	4.97±0.17aE	11.04±0.43bD	15.88±1.45bC	18.90±1.45bB	28.43±2.72bA
	GR	4.85±0.12aE	9.92±1.29bcD	14.74±0.95bC	18.17±2.21bB	25.26±1.62bA
	TPGR	5.12±0.26aE	7.06±0.43cD	10.82±0.69cC	14.10±0.80cB	19.51±1.30aA
CAD	Control	7.17±0.82aE	12.98±1.97aD	18.84±0.88aC	28.96±0.39aB	43.28±1.80aA
	TP	7.50±0.35aD	8.78±0.61bD	11.09±0.65bC	14.53±0.77bB	19.27±1.62bA
	GR	7.34±0.21aD	9.16±0.58bC	12.15±0.53bB	13.25±1.32bB	17.68±1.18bcA
	TPGR	7.47±0.04aD	8.44±0.36bD	11.05±0.78bC	13.86±0.65bB	16.09±0.75cA
HIM	Control	17.09±1.15aE	27.85±1.72aD	49.35±2.02aC	74.32±2.84aB	113.37±5.24aA
	TP	17.42±2.08aE	23.12±1.47bD	27.83±0.46bC	34.06±1.17bB	41.88±0.69bA
	GR	17.51±0.61aE	22.98±1.39bD	25.12±0.17cC	30.56±2.60bB	36.09±1.65cA
	TPGR	17.95±0.68aE	19.57±0.81cD	22.66±1.16dC	26.36±0.41cB	30.73±0.55dA
PHE	Control	2.56±0.27aE	7.93±0.22aD	16.04±1.60aC	24.93±0.34aB	35.88±1.27aA
	TP	2.53±0.27aE	4.61±0.38bD	6.63±0.35bC	9.41±0.26bB	14.80±0.62bA
	GR	2.59±0.36aE	4.38±0.21bD	6.69±0.63bC	9.38±0.30bB	14.70±1.34bA
	TPGR	2.68±0.30aE	3.62±0.40cD	5.45±0.33bC	7.44±0.18cB	11.42±0.15cA
TRY	Control	8.02±0.43aE	21.21±1.66aD	37.76±1.88aC	57.01±2.59aB	85.26±1.95aA
	TP	7.89±0.48aE	14.83±1.62bD	23.01±2.62bC	30.91±0.32bB	37.46±1.37bA
	GR	7.93±0.62aE	14.85±1.93bD	24.08±1.06bC	30.99±1.03bB	39.21±1.22bA
	TPGR	8.00±0.75aE	10.76±0.43cD	17.41±1.19cC	25.34±0.93cB	33.25±0.56cA
TYR	Control	ND	17.10±2.08aD	44.05±2.31aC	67.76±2.36aB	95.80±4.15aA
	TP	ND	13.16±1.54bcD	17.49±1.04bC	28.02±0.59bB	38.52±0.99bA
	GR	ND	13.55±0.31bD	17.15±1.02bcC	27.66±0.47bB	35.47±1.33bcA
	TPGR	ND	10.80±0.57cD	14.55±0.91cC	23.14±1.39cB	32.72±1.74cA
SPD	Control	ND	ND	ND	ND	ND
	TP	ND	ND	ND	ND	ND
	GR	ND	ND	ND	ND	ND
	TPGR	ND	ND	ND	ND	ND
SPM	Control	ND	ND	ND	ND	ND
	TP	ND	ND	ND	ND	ND
	GR	ND	ND	ND	ND	ND
	TPGR	ND	ND	ND	ND	ND

*PUT, putrescine; CAD, cadaverine; HIM, histamine; PHE, 2-phenylethylamine; TRY, tryptamine; TYR, tyramine; SPM, spermine; SPD, spermidine; ND, not detected. Values are the mean of three replications ± standard deviation. Different small letters in the same column indicate significant differences between means (p < 0.05). Different capital letters in the same row indicate significant differences between means (p < 0.05)*.

### Effect of tea polyphenols in combination with 6-gingerol treatment on sensory evaluation

Results in Figure 2B revealed the sensory changes of shrimp paste with different treatments during 25°C storage. Color, aroma, flavor and juiciness of control samples were given “unacceptable” scores by the end of storage period. The results of sensory evaluation were correlated with TVC and chemical analyses. The antioxidant and antimicrobial effects of TPGR had been shown to prolong the shelf life of shrimp paste by 40–60 days as compared to the control samples.

## Conclusion

The effect of tea polyphenols combined with 6-gingerol (TPGR) on the biogenic amines inhibition and quality of shrimp paste stored at 25°C for 160 days was investigated. Shrimp paste color, total volatile basic nitrogen, thiobarbituric acid reactive substances, total amino acids, biogenic amines, microbial, and sensory quality were measured. Our research exhibited that the quality preservation of ambient-stored shrimp paste by TPGR treatment involved the maintenance of color, total amino acids content and sensory quality, reduction of microbial counts, inhibition of biogenic amines compared with control. TPGR samples also showed lower levels of TVB-N and TBA value during storage. These results indicate that TPGR is promising as a synergistic preservative to be used for maintaining fish and fish products quality and extending their shelf-life.

### Conflict of interest statement

The authors declare that the research was conducted in the absence of any commercial or financial relationships that could be construed as a potential conflict of interest.

## References

[B1] Afzal M. Safer A. M. Menon M. (2015). Green tea polyphenols and their potential role in health and disease. Inflammopharmacology, 23, 151–161. 10.1007/s10787-015-0236-1 26164000

[B2] Baliga M. S. Haniadka R. Pereira M. M. Thilakchand K. R. Rao S. Arora R. (2012). Radioprotective effects of *Zingiber officinale* Roscoe (Ginger): past, present and future. Food Funct. 3, 714–723. 10.1039/c2fo10225k 22596078

[B3] Bandyopadhyay M. Chakraborty R. Raychaudhuri U. (2008). Antioxidant activity of natural plant sources in dairy dessert (Sandesh) under thermal treatment. LWT Food Sci. Technol. 41, 816–825. 10.1016/j.lwt.2007.06.001

[B4] Botsoglou N. A. Fletouris D. J. Papageorgiou G. E. Vassilopoulos V. N. Mantis A. J. Trakatellis A. G. (1994). Rapid, sensitive and specific thiobarbituric acid method for measuring peroxidation in animal tissue, food and feedstuff samples. J. Agric. Food Chem. 42, 1931–1937. 10.1021/jf00045a019

[B5] Bueno-Solano C. Lopez-Cervantes J. Campas-Baypoli O. N. Lauterio-Garcia R. Adan-Bante N. P. Sanchez-Machado D. I. (2009). Chemical and biological characteristics of protein hydrolysates from fermented shrimp by-products. Food Chem. 112, 671–675. 10.1016/j.foodchem.2008.06.029

[B6] Cai L. Y. Cao A. L. Li Y. C. Song Z. Leng L. P. Li J. R. (2015a). The effects of essential oil treatment on the biogenic amines inhibition and quality preservation of red drum (*Sciaenops ocellatus*) fillets. Food Control 56, 1–8. 10.1016/j.foodcont.2015.03.009

[B7] Cai L. Y. Wang Y. B. Cao A. L. Lv Y. F. Li J. R. (2015b). Effect of alginate coating enriched with 6-gingerol on the shelf life and quality changes of refrigerated red sea bream (*Pagrosomus major*) fillets. RSC Adv. 5, 36882–36889. 10.1039/C5RA04551G

[B8] Cai L. Wu X. Dong Z. Li X. Yi S. Li J. (2014). Physicochemical responses and quality changes of red sea bream (*Pagrosomus major*) to gum arabic coating enriched with ergothioneine treatment during refrigerated storage. Food Chem. 160, 82–89. 10.1016/j.foodchem.2014.03.093 24799212

[B9] Chinnathambi A. Alharbi S. A. (2013). Assessement of the antimicrobial and antioxidant activities of green tea and black tea. J. Pure Appl. Microbiol. 7, 2691–2696.

[B10] Cross H. R. Moen R. Stanfield M. S. (1978). Training and testing of judges for sensory analysis of meat quality. Food Technol. 32, 48–54.

[B11] Dangles O. (2012). Antioxidant activity of plant phenols: chemical mechanisms and biological significance. Curr. Org. Chem. 16, 692–714. 10.2174/138527212799957995

[B12] Faithong N. Benjakul S. (2014). Changes in antioxidant activities and physicochemical properties of Kapi, a fermented shrimp paste, during fermentation. J. Food Sci. Technol. 51, 2463–2471. 10.1007/s13197-012-0762-4 25328185 PMC4190242

[B13] Feng L. F. Jiang T. J. Wang Y. B. Li J. R. (2012). Effects of tea polyphenol coating combined with ozone water washing on the storage quality of black sea bream (*Sparus macrocephalusi*). Food Chem. 135, 2915–2921. 10.1016/j.foodchem.2012.07.078 22980890

[B14] Izquierdo Canas P. M. Garcia Romero E. Gomez Alonso S. Fernandez Gonzalez M. Palop Herreros M. L. L. (2008). Amino acids and biogenic amines during spontaneous malolactic fermentation in Tempranillo red wines. J. Food Composition Anal. 21, 731–735. 10.1016/j.jfca.2007.11.002

[B15] Jeena K. Liju V. B. Viswanathan R. Kuttan R. (2014). Antimutagenic potential and modulation of carcinogen-metabolizing enzymes by ginger essential oil. Phytother. Res. 28, 849–855. 10.1002/ptr.5064 24023002

[B16] Kim H. S. Lee S. H. Byun Y. Park H. D. (2015). 6-Gingerol reduces Pseudomonas aeruginosa biofilm formation and virulence via quorum sensing inhibition. Sci. Rep. 5, 8656. 10.1038/srep08656 25728862 PMC4345325

[B17] Kim J. S. Shahidi F. Heu M. S. (2003). Characteristics of salt-fermented sauces from shrimp processing byproducts. J. Agric. Food Chem. 51, 784–792. 10.1021/jf020710j 12537458

[B18] Kumudavally K. V. Phanindrakumar H. S. Tabassum A. Radhakrishna K. Bawa A. S. (2008). Green tea—a potential preservative for extending the shelf life of fresh mutton at ambient temperature (25 ± 2°C). Food Chem. 107, 426–433. 10.1016/j.foodchem.2007.08.045

[B19] Kuriyama S. Shimazu T. Ohmori K. Kikuchi N. Nakaya N. Nishino Y.. (2006). Green tea consumption and mortality due to cardiovascular disease, cancer, and all causes in Japan—The ohsaki study. JAMA 296, 1255–1265. 10.1001/jama.296.10.1255 16968850

[B20] Li T. Li J. Hu W. Li X. (2013). Quality enhancement in refrigerated red drum (*Sciaenops ocellatus*) fillets using chitosan coatings containing natural preservatives. Food Chem. 138, 821–826. 10.1016/j.foodchem.2012.11.092 23411183

[B21] Mah J. H. Kim Y. J. Hwang H. J. (2009). Inhibitory effects of garlic and other spices on biogenic amine production in Myeolchi-jeot, Korean salted and fermented anchovy product. Food Control 20, 449–454. 10.1016/j.foodcont.2008.07.006

[B22] Moon J. S. Kim Y. Jang K. I. Cho K. J. Yang S. J. Yoon G. M. (2010). Analysis of biogenic amines in fermented fish products consumed in Korea. Food Sci. Biotechnol. 19, 1689–1692. 10.1007/s10068-010-0240-6

[B23] Nile S. H. Park S. W. (2015). Chromatographic analysis, antioxidant, anti-inflammatory, and xanthine oxidase inhibitory activities of ginger extracts and its reference compounds. Ind. Crops Prod. 70, 238–244. 10.1016/j.indcrop.2015.03.033

[B24] Ozogul Y. Balikci E. (2013). Effect of various processing methods on quality of mackerel (*Scomber scombrus*). Food Bioprocess Technol. 6, 1091–1098. 10.1007/s11947-011-0641-4

[B25] Park J. S. Lee C. H. Kwon E. Y. Lee H. J. Kim J. Y. Kim S. H. (2010). Monitoring the contents of biogenic amines in fish and fish products consumed in Korea. Food Control 21, 1219–1226. 10.1016/j.foodcont.2010.02.001

[B26] Pasrija D. Ezhilarasi P. N. Indrani D. Anandharamakrishnan C. (2015). Microencapsulation of green tea polyphenols and its effet on incorporated bread quality. LWT Food Sci. Technol. 64, 289–296. 10.1016/j.lwt.2015.05.054

[B27] Shariatpanahi Z. V. Taleban F. A. Mokhtari M. Shahbazi S. (2010). Ginger extract reduces delayed gastric emptying and nosocomial pneumonia in adultrespiratory distress syndrome patients hospitalized in an intensive care unit. J. Crit. Care 25, 647–650. 10.1016/j.jcrc.2009.12.008 20149584

[B28] Singh G. Kapoor I. P. Singh P. de Heluani C. S. de Lampasona M. P. Catalan C. A. (2008). Chemistry, antioxidant and antimicrobial investigations on essential oil and oleoresins of *Zingiber officinale*. Food Chem. Toxicol. 46, 3295–3302. 10.1016/j.fct.2008.07.017 18706468

[B29] Stoilova I. Krastanov A. Stoyanova A. Denev P. Gargova S. (2007). Antioxidant activity of a ginger extract (*Zingiber officinale*). Food Chem. 102, 764–770. 10.1016/j.foodchem.2006.06.02326283843

[B30] Wagner H. (2011). Synergy research: approaching a new generation of phytopharmaceuticals. Fitoterapia 82, 34–37. 10.1016/j.fitote.2010.11.016 21075177

[B31] Yusof K. M. Makpol S. Jamal R. Harun R. Mokhtar N. Ngah W. Z. W. (2015). Gamma-tocotrienol and 6-gingerol in combination synergistically induce cytotoxicity and apoptosis in HT-29 and SW837 human colorectal cancer cells. Molecules 20, 10280–10297. 10.3390/molecules200610280 26046324 PMC6272690

[B32] Zhao J. Lv W. Wang J. Li J. Liu X. Zhu J. (2013). Effects of tea polyphenols on the post-mortem integrity of large yellow croaker (*Pseudosciaena crocea*) fillet proteins. Food Chem. 141, 2666–2674. 10.1016/j.foodchem.2013.04.126 23871009

